# Theaflavin −3,3'-digallate/ethanol: a novel cross-linker for stabilizing dentin collagen

**DOI:** 10.3389/fbioe.2024.1401032

**Published:** 2024-05-15

**Authors:** Zhiyong Chen, Yingxian Wei, Likun Liang, Xu Wang, Fangfei Peng, Yiying Liang, Xin Huang, Kaiqi Yan, Yunxia Gao, Kangjing Li, Xiaoman Huang, Xinglu Jiang, Wenxia Chen

**Affiliations:** ^1^ Guangxi Key Laboratory of Oral and Maxillofacial Rehabilitation and Reconstruction, Guangxi Clinical Research Center for Craniofacial Deformity, College & Hospital of Stomatology, Guangxi Medical University, Nanning, China; ^2^ Department of Prosthodontics, College & Hospital of Stomatology, Guangxi Medical University, Nanning, China; ^3^ Department of Endodontics, College & Hospital of Stomatology, Guangxi Medical University, Nanning, China; ^4^ Clinical Laboratory Medicine Department, College & Hospital of Stomatology, Guangxi Medical University, Nanning, China

**Keywords:** theaflavin-3,3’-digallate, dentin collagen, cross-linking, collagenase digestion, mechanism

## Abstract

**Objectives:**

To study the ability of theaflavin-3,3’-digallate (TF3)/ethanol solution to crosslink demineralized dentin collagen, resist collagenase digestion, and explore the potential mechanism.

**Methods:**

Fully demineralized dentin blocks were prepared using human third molars that were caries-free. Then, these blocks were randomly allocated into 14 separate groups (n = 6), namely, control, ethanol, 5% glutaraldehyde (GA), 12.5, 25, 50, and 100 mg/ml TF3/ethanol solution groups. Each group was further divided into two subgroups based on crosslinking time: 30 and 60 s. The efficacy and mechanism of TF3’s interaction with dentin type I collagen were predicted through molecular docking. The cross-linking, anti-enzymatic degradation, and biomechanical properties were studied by weight loss, hydroxyproline release, scanning/transmission electron microscopy (SEM/TEM), *in situ* zymography, surface hardness, thermogravimetric analysis, and swelling ratio. Fourier transform infrared spectroscopy (FTIR), X-ray photoelectron spectroscopy (XPS), and Raman spectroscopy were utilized to explore its mechanisms. Statistical analysis was performed using one and two-way analysis of variance and Tukey’s test.

**Results:**

TF3/ethanol solution could effectively crosslink demineralized dentin collagen and improve its resistance to collagenase digestion and biomechanical properties (*p* < 0.05), showing concentration and time dependence. The effect of 25 and 50 mg/ml TF3/ethanol solution was similar to that of 5% GA, whereas the 100 mg/mL TF3/ethanol solution exhibited better performance (*p* < 0.05). TF3 and dentin type I collagen are mainly cross-linked by hydrogen bonds, and there may be covalent and hydrophobic interactions.

**Conclusion:**

TF3 has the capability to efficiently cross-link demineralized dentin collagen, enhancing its resistance to collagenase enzymatic hydrolysis and biomechanical properties within clinically acceptable timeframes (30 s/60 s). Additionally, it exhibits promise in enhancing the longevity of dentin adhesion.

## 1 Introduction

The basis of dentin bonding lies in the hybrid layer, which is formed when the adhesive penetrates and intertwines with the intricate network of demineralized dentin collagen fibers (Anastasiadis et al., 2021). However, achieving optimal infiltration poses a challenge, often leaving collagen fibers exposed within the hybrid layer and compromising the structural integrity and stability of the dentin bonding interface (Coutinho et al., 2011). Over the long term, these exposed collagen fibers are susceptible to degradation by bacterial enzymes and endogenous dentin collagenases (matrix metalloproteinases, MMPs, and cysteine cathepsins, CCs). This enzymatic hydrolysis undermines the hybrid layer’s stability, ultimately leading to a significant reduction in adhesive durability (Fan-Chiang et al., 2023).

For an extended period, scientific research has primarily focused on enhancing the quality of the hybrid layer and bolstering the longevity of the adhesive (Van Meerbeek et al., 2020). For example, through biomimetic remineralization, the exposed collagen fibers are remineralized (Niu et al., 2017; Ma et al., 2021). By applying inhibitors of MMPs and CCs, the enzymatic digestion process of collagen fibers is inhibited (Comba et al., 2019). The physical and chemical characteristics of collagen are optimized through cross-linking, which hides the binding sites of enzymes, thus increasing its resistance to enzymatic hydrolysis (Wang et al., 2022) and so on. Despite these enhancements, issues like biosafety, cumbersome operation, limited durability and ambiguous long-term efficacy persist, leading to no agreed-upon view regarding dentin bonding.

Lately, as it becomes more clearly that maintaining the natural cross-linking condition of dentin collagen is important, attention shifts to stabilizing demineralized dentin collagen through cross-linking, giving rise to the ‘dentin biomodification’ concept (Cai et al., 2018). Currently, the dentin biomodification is often carried out by chemical synthesis and natural crosslinking agents. Among them, glutaraldehyde (GA) is recognized as a highly efficient chemical crosslinking agent, which is the ’ gold standard ’ of crosslinking agents and is commonly used for tissue fixation. However, GA has strong cytotoxicity and its clinical application is limited (Cilli and Prakki, 2009; Cai et al., 2018). Therefore, natural polyphenolic cross-linking agents derived from plants have attracted more and more attention due to their strong and low toxicity, such as proanthocyanidins (PA), epigallocatechin gallate (EGCG), etc (Zhao et al., 2022). Studies have shown that naturally occurring polyphenolic compounds can interact with collagen fibers through phenolic hydroxyl and galloyl groups to form covalent, ionic, and hydrogen bonds. They can also alter the spatial conformation of collagen, form cross-links with endogenous MMPs or CCs, obstruct enzyme recognition sites, enhance the mechanical properties of collagen, lessen its sensitivity to enzymes, and locally create a hydrophobic microenvironment that lowers water absorption and swelling ratio (Liu et al., 2015; Reis et al., 2021; Chiang et al., 2023). As mentioned above, recent studies have highlighted the potential of natural polyphenols in dentin biomodification, with PA being particularly noted for its efficacy in this domain (Wang B. et al., 2023). Despite PA’s potential in enhancing dentin properties, it is not the ideal collagen cross-linking agent due to limitations: complex molecular structure (Anumula et al., 2022), high molecular weight and low penetration efficiency (Hass et al., 2021), and affecting the curing of the adhesive by scavenging free radicals (Gabetta et al., 2000; Green et al., 2010), plus its dark color causes staining (Gabetta et al., 2000).

Given this, scholars are still committed to finding better natural collagen crosslinking agents. In recent years, the research on theaflavins (TFs) has been increasing due to their ability to crosslink with collagen and produce excellent biological effects, and other biological activities like anti-cancer, anti-bacterial and anti-inflammatory (Vidal et al., 2016; Hass et al., 2021; Guo et al., 2023). TFs is mainly composed of four dimers, namely, theaflavin (TF1), theaflavin-3-gallate (TF2A), theaflavin-3’-gallate (TF2B), and theaflavin-3,3’-digallate (TF3). These four dimers differ from each other based on the number and positioning of the connected gallic acyl groups. However, most of current studies are aimed at TFs, and the role of the four dimers in it is still unknown.

Preliminary studies suggest that among the four dimers of the TFs, TF3 has the largest number of phenolic hydroxyl groups and gallic acyl groups and the most crosslinking potential (Takemoto and Takemoto, 2018). Regrettably, no research has been conducted to assess how TF3 interacts with demineralized dentin collagen and to understand its underlying mechanism. Therefore, our research studied the cross-linking ability of TF3, for the very first time, used in demineralized dentin collagen. Furthermore, we explored the characteristics of TF3, including resistance to internal and external collagenase digestion, biomechanical properties, and its potential mechanism of action, to provide an efficient and clinically feasible new method for improving the longevity of the adhesive. The null hypotheses tested were that TF3 cannot 1) cross-link demineralized dentin collagen by chemical action, 2) enhance the enzymatic hydrolysis resistance of demineralized dentin collagen to internal and external collagenases, or 3) improve the biomechanical characteristics of demineralized dentin collagen.

## 2 Materials and methods

### 2.1 Chemicals and reagents

**FIGURE 1:**
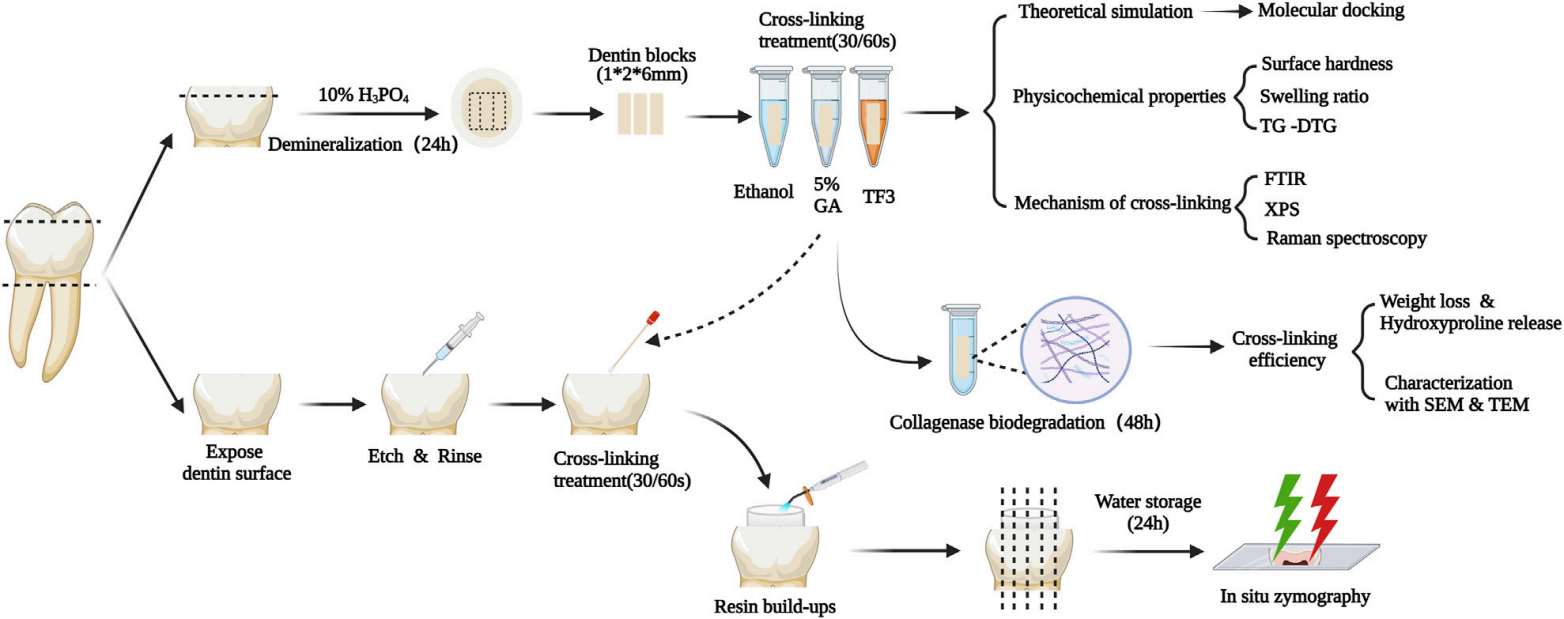
Schematic diagram of the experimental design. TG-DTG: Thermogravimetric Analysis and Differential Thermal Gravity. FTIR: Fourier transformed infrared spectroscopy. XPS: X-ray photoelectron spectroscopy. SEM&TEM: Scanning/Transmission electron microscopy.

TF3 was purchased from Chengdu Biopurify Phytochemicals Ltd. (Chengdu, China). Ethanol, 25% GA, and phosphate buffer saline were bought from Aladdin (Shanghai, China). 85% phosphoric acid was bought from Shanghai Acmec Biochemical Co., Ltd. (Shanghai, China). Acetic acid was bought from CHRON (Chengdu, China). Ammonium bicarbonate was bought from Macklin (Shanghai, China). 35% Phosphoric-acid gel, Adaper TM Single bond 2 Adhesive, and Resin composite Filtek TM Z250 were bought from 3 M (St. Paul, United States). Figure 1 illustrates the workflow of the experimental process.

### 2.2 Preparation of solutions

At room temperature and dark conditions, dissolve TF3 in ethanol to prepare TF3 solutions with concentrations of 12.5 mg/mL, 25 mg/mL, 50 mg/mL, and 100 mg/mL. Meanwhile, dilute 25% GA to 5% GA using ethanol. After preparation, dispense 0.5 mL solution into 1.5 mL light-proof Eppendorf tubes for future use. Also, under room temperature and dark conditions, prepare a 0.1 mg/mL type I collagenase solution using ammonium bicarbonate buffer. Adjust the pH to 7.1–7.2 by adding glacial acetic acid drop by drop, then weigh and add type I collagenase to the solution, and measure the pH again. All solutions should be prepared and used immediately.

### 2.3 Theoretical simulation

#### 2.3.1 Molecular docking.

**FIGURE 2:**
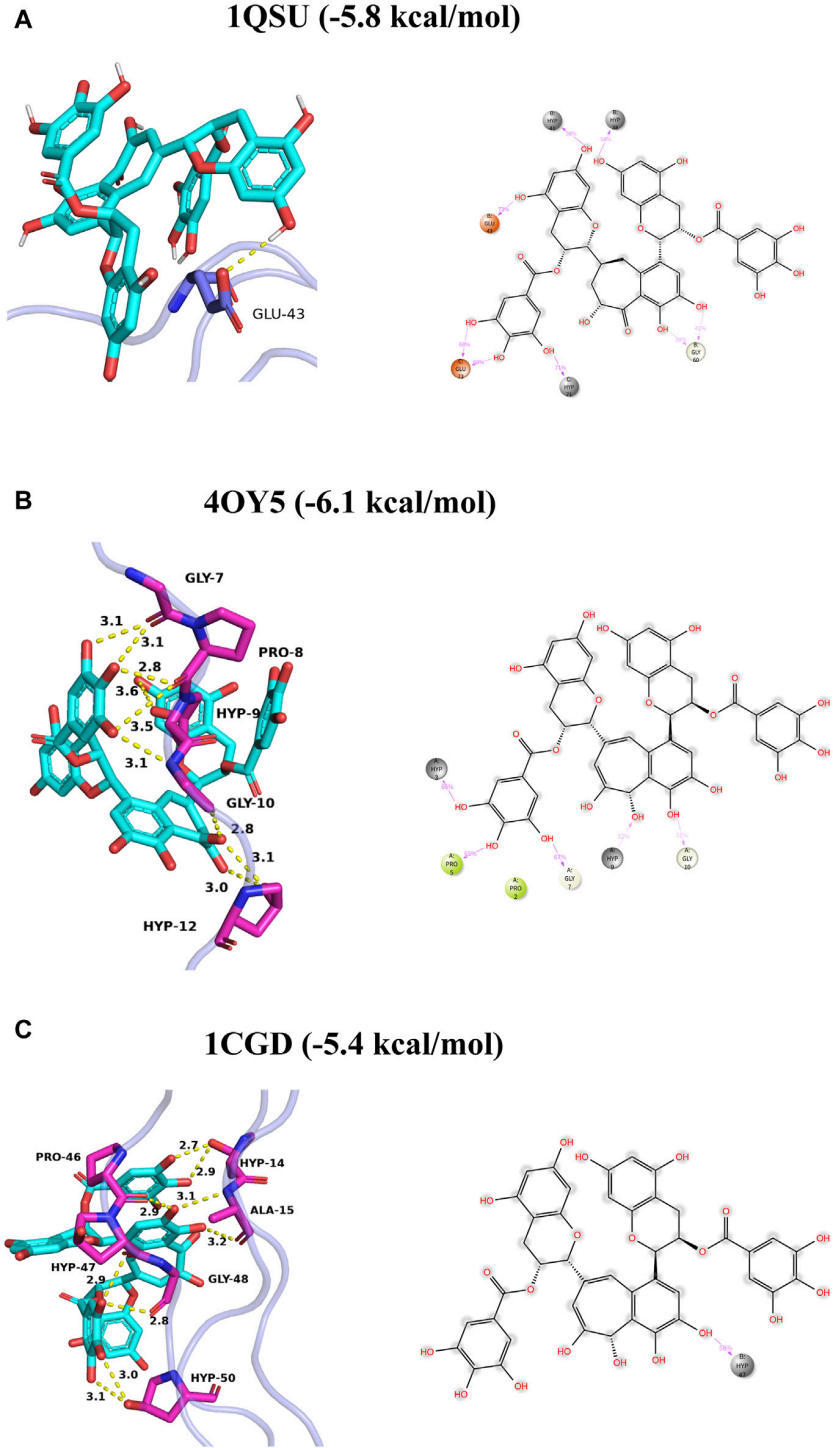
Binding mode of TF3 to collagen I **(A–C)**. The left panel shows three-dimensional plots, where hydrogen bonds are depicted by yellow dashed lines and hydrophobic interactions are shown by green dashed lines. The right panel shows two-dimensional plots, where the magenta solid line represents the ion action.

Collagen’s X-ray crystallographic formations (PDB: 1QSU, 4OY5, 1CGD) were obtained from the Protein Data Bank. Each compound’s protonation state was set to a pH of 7.4, followed by their expansion to three-dimensional structures via Open Babel. The receptor protein and ligands were processed and parameterized using AutoDock Tools (ADT3). Documentation for the docking grid was produced by the Auto Grid of sitemap, and Auto Dock Vina (1.2.0) was utilized for the docking simulation. The most suitable pose was chosen for interaction analysis. Ultimately, PyMOL was responsible for creating the protein-ligand interaction as depicted in Figure 2.

### 2.4 Cross-linking efficiency

#### 2.4.1 Preparation of experimental materials

Following the acquisition of informed consent from the donors, ninety non-carious human third molars were gathered, adhering to the protocols approved the Ethics Committee for Human Studies of the College and Hospital of Stomatology, Guangxi Medical University. Before usage, the teeth were preserved in the 0.1% thymol solution at a temperature of 4°C for 1 month.

The crown’s occlusal segment and the adjacent enamel walls were surgically excised with the aid of a precision low-speed diamond saw equipped with a water-cooling mechanism (EXAKT E300CP, Germany) to obtain dentin blocks. Afterward, 85% phosphoric acid mixed with deionised water to form 10% phosphoric acid, and dentin blocks were placed in a 10% phosphoric acid solution and shaken in a 25°C constant temperature shaking table for 24 h to ensure them were demineralized completely. Subsequently, the demineralized dentin blocks underwent a rigorous washing process, being thoroughly cleansed with distilled water three times, followed by a 1-h hydration period. Finally, these demineralized dentin blocks were cut into 1 × 2 × 6 mm.

#### 2.4.2 Weight loss (WL) and hydroxyproline release (HYP)

The demineralized dentin blocks were randomly allocated into 14 distinct groups, with 6 blocks in each group. At room temperature, the demineralized dentin blocks were cross-linked with 0.5 mL of 12.5 mg/mL, 25 mg/mL, 50 mg/mL, and 100 mg/mL of TF3, 5% GA, and ethanol solutions, respectively, for 30 s or 60 s. Following the cross-linking, the blocks underwent a rigorous cleaning process involving three washes with distilled water. Subsequently, they were subjected to drying in a freeze-vacuum dryer for 12 h to achieve complete dehydration. Three measurements were taken of the demineralized dentin block’s mass by electronic balance, with the mean measurement being documented as M0. Subsequently, the dentin blocks underwent hydration using distilled water for 1 hour, followed by immersion in 1 mL of 0.1 mg/mL type I collagenase solution (Sigma-Aldrich, St. Louis, MO, USA) and digestion in a 37°C thermostatic shaker for 48 h. After the end of enzymatic hydrolysis, cleaning, drying, and weighing again, it was recorded as M1. The weight loss rate (d = 0.01 mg) was calculated as per this equation:

$$\text{WL }\%=\left[\left(M0-M1\right)/M0\right]\times 100\%.$$

The 0.5 mL enzymatic hydrolysate of collagen from each group was utilized for the determination of HYP release, with reference to the detailed methodology provided in the hydroxyproline kit instructions (Nanjing Jiancheng Bioengineering Institute, Nanjing, China). Subsequently, the release of HYP was calculated by measuring the absorbance at 550 nm using a microplate reader.

#### 2.4.3 Scanning electron microscope (SEM)

After cross-linked in 100 mg/ml TF3, 5% GA, and ethanol solutions, the demineralized dentin blocks were hydrolyzed by collagenase solution, and then underwent overnight fixation in 2.5% glutaraldehyde. Subsequently, they were dehydrated using ethanol solutions of 30%, 50%, 70%, 80%, 90%, and 95%, with each step lasting 15 min. This was followed by two successive 20-min immersions in 100% ethanol. The specimens underwent a 30-min treatment using a blend of ethanol and isoamyl acetate (V/V = 1/1), followed by a 1-h or overnight exposure to solely isoamyl acetate. Subsequent to undergoing critical point drying, the specimens’ morphology was meticulously examined utilizing a biological scanning electron microscope (Hitachi SU3800, gold-sprayed by Hitachi MC1000, Japan).

#### 2.4.4 Transmission electron microscopy (TEM)

After cross-linked in 100 mg/ml TF3, 5% GA, and ethanol solutions, the demineralized dentin blocks were hydrolyzed by collagenase solution, and then underwent overnight fixation in 2.5% glutaraldehyde, dehydration using a series of ethanol solutions, and subsequent embedding in epoxy resin as per established guidelines. The specimens were sectioned by Ultramicrotome (EMUC7, Leica Microsystems, Germany) and observed under TEM (Tecnai G2 Spirit, FEI, USA). The accelerating voltage was 80 kV.

### 2.5 Physicochemical properties

#### 2.5.1 Surface hardness test

According to the above method, 1 mm dentin blocks were prepared, which were cross-linked in 100 mg/ml TF3, 5% GA, and ethanol solutions respectively. Their surfaces were etched using a 35% phosphoric-acid gel for 15 s. Afterward, the blocks were thoroughly rinsed with deionized water and dried. Subsequently, the samples in each group (n = 6) were cross-linked for 30 s and vacuum freeze-dried for 12 h. After being thoroughly dried, observing under a microscope, indentations were produced in the middle of the dentin blocks with a flat surface. Then, the surface Vickers hardness was calculated by loading 15 s at a weight of 50 g with the same indentation process.

#### 2.5.2 Swelling ratio test

At room temperature, demineralized dentin collagen (n = 6) which were cross-linked in 100 mg/ml TF3, 5% GA, and ethanol solutions respectively, were swollen in distilled water and balanced overnight in phosphate buffer. The specimens were immediately weighed after drying with filter paper, which was recorded as wet weight M0. After removing the phosphate by soaking the samples in distilled water for 10 min, they were subjected to vacuum freeze-drying for 12 h. Following this, the specimens were re-weighed and recorded as dry weight M1. This equation is used to compute the swelling ratio:

$$\text{Swelling ratio}=\left[\left(M0-M1\right)/M1\right]\times 100\;\%$$

#### 2.5.3 Thermogravimetric Analysis and Differential Thermal Gravity (TG-DTG)

The relationship between weight loss and temperature after cross-linking in 100 mg/ml TF3, 5% GA, and ethanol solutions respectively of demineralized dentin was measured by a thermogravimetric analyzer (Netzsch STA-2500), including TG and DTG curves. The parameters are set in the following manner: the nitrogen flow rate is 10 mL/min and the temperature rises to 200°C. Then, the test begins after falling to room temperature. Following this, the temperature rises to 1,000°C at a heating rate of 10°C/min.

### 2.6 Enzyme inhibition

#### 2.6.1 *In situ* zymography

The enamel of the tooth crown was removed and the dentin was exposed by a low-speed water-cooled diamond saw (EXAKT E300CP Germany). According to the total acid etching dentin bonding process, the dentin surface was treated with acid etching, washing, and drying. Subsequently, the crosslinking agent solutions, which were 100 mg/ml TF3, 5% GA, and ethanol solutions respectively, were applied for 30 s on the dentin surface, following by washing and drying. Then, the Adaper TM Single bond 2 adhesive was evenly applied, blow-thinned and cured, and filled with 2 mm resin composite (Filtek TM Z250). Finally, the samples were immersed in distilled water at 37°C for 24 h. After that, the samples from each group were cut using a low-speed water-cooled diamond saw parallel to the long axis of the tooth, obtaining 1 mm thick tooth slices. Subsequently, the samples were adhered to glass slides and polished wet with silicon carbide papers of 600, 1,200, and 2000 grit sizes in sequence. The final specimens, together with the coverslip, were around 1.13 mm thick. According to the operation steps of the EnzChek Gelatinase/Collagenase Assay Kit (Invitrogen, Carlsbad, United States), Gelatin was diluted using phosphate buffer saline, and 50ul gelatin was dripped onto each specimen before covering with a coverslip to seal the edges. The activity of matrix metalloproteinases in the hybrid layer (λex/λem = 494 nm/521 nm) was observed by confocal laser scanning microscopy (Fluoview FV3000; Olympus, Tokyo, Japan).

### 2.7 Mechanism of cross-linking

#### 2.7.1 Fourier transform infrared spectroscopy (FTIR)

TF3 powder and demineralized dentin matrixs treated with 100 mg/ml TF3, 5% GA, and ethanol solutions were freeze-dried and then investigated by Fourier transform infrared spectrometer (Thermo Scientific iN10, USA). The spectra were recorded in the wavelength range of 400–4,000 cm^−1,^ with a resolution of 4 cm^−1^, to capture the detailed vibrational modes of the molecules present. The obtained spectra were analyzed by Thermo Scientific^TM^OMNIC™ software.

#### 2.7.2 X-ray photoelectron spectroscopy (XPS)

The demineralized dentin matrixs treated with 100 mg/ml TF3, 5% GA, and ethanol solutions were freeze-dried and placed in the sample chamber of the XPS instrument (Thermo Scientific K-Alpha, USA). When the sample chamber pressure is lower than 2.0 × 10^-7^mbar, the sample is sent to the analysis chamber. The parameters are set as follows: the spot size is 400 μm, the operating voltage is 12 kV, the filament current is 6 mA, the full spectrum scanning energy is 150 eV, and the step size is 1 eV. Fine spectral scanning of carbon, nitrogen and oxygen elements was carried out with a scanning energy of 50 eV and a step size of 0.1 eV.

#### 2.7.3 Raman spectroscopy

TF3 powder and demineralized dentin collagen treated with 100 mg/ml TF3, 5% GA, and ethanol solutions were freeze-dried and analyzed by Raman spectrometer (Horiba Lab RAM HR Evolution, Japan). The parameters are set as follows: The sample is divided into 1 mm blocks, and three scans lasting 60 s are performed in the specified sample area. Details of the spectrum are captured using a laser beam with a wavelength of 514 nm, ranging from 50 cm−^1^–4,000 cm−^1^.

### 2.8 Statistical analysis

Data on weight loss, hydroxyproline release and swelling ratio were evaluated using two-factor analysis of variance (ANOVA), followed by Tukey’s *post hoc* test. The two factors were the solution (factor 1) and treatment duration (factor 2). Data on surface hardness was evaluated using one-factor analysis of variance (ANOVA) and Tukey’s *post hoc* test. The significance level was set to 0.05. Statistical analysis was performed using GraphPad Prism 9 (GraphPad Software, San Diego, California, U.S.).

## 3 Results

### 3.1 Molecular docking

Grounded in the hypothesis that minimal energy is the best-fitting model, the molecular docking of TF3 with type I collagen was simulated. The binding energy and predicted interaction are shown in Table 1 and binding mode of TF3 to collagen I are shown in Figure 2.

**TABLE 1:** Predicted interactions of the type I Collagen binding-site Residues with TF3.

Type I Collagen	Binding energy (kcal/mol)	Predicted interactions
1QSU	−5.8	Glu-43, Glu73
4OY5	−6.1	Gly7
1CGD	−5.4	Hyp47

### 3.2 Cross-linking efficiency

#### 3.2.1 Weight loss and hydroxyproline release

**FIGURE 3:**
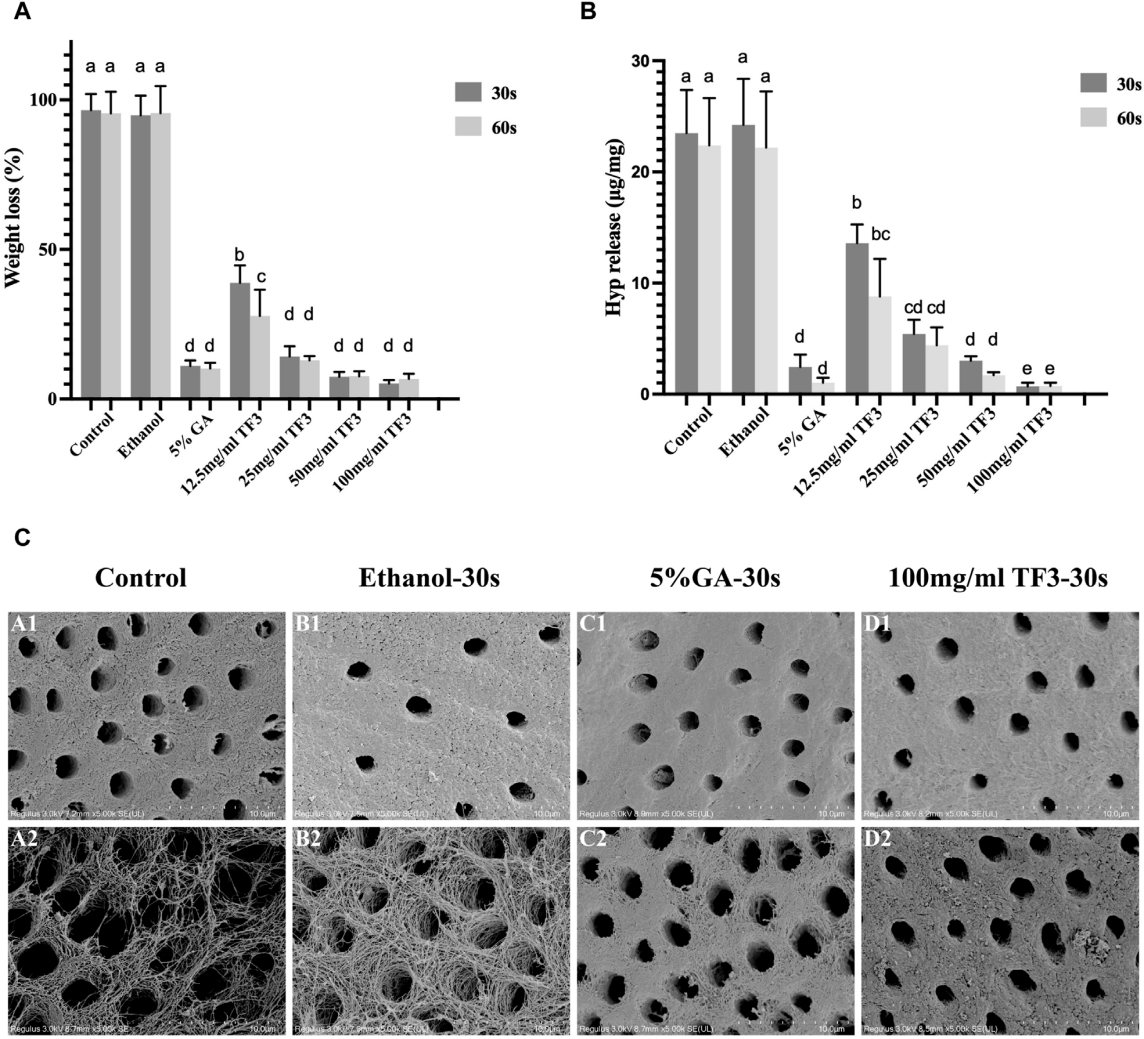
Weight loss **(A)** and hydroxyproline release **(B)** of demineralized dentin matrix with different cross-linkers. Variations in lowercase letters signify notable disparities among the groups (*p* < 0.05). Representative SEM images of demineralized dentin matrix with diverse treatments **(C)**: A1 to D1 represent before digestion and A2 to D2 represent after digestion. The scale bar is set at 10 um.

It was suggested that WL rate (Figure 3A) of different TF3 concentrations of 12.5 mg/mL, 25 mg/mL, 50 mg/mL, 100 mg/mL group, and GA group were lower than that of the control and ethanol group (*p* < 0.05). Additionally, between the control group and the ethanol group, there existed no significant difference (*p* > 0.05).

The results of WL rate echoed those of HYP release. There was no difference in HYP release (Figure 3B) between the ethanol group and the control group (*p* > 0.05). With TF3 concentrations increased, the figure of HYP release steadily got close to the positive control GA group. The HYP release showed no significant difference between the 50 mg/ml TF3 and GA groups (*p* > 0.05). The HYP release of 100 mg/ml TF3 group was the lowest in all groups, which was statistically less than the GA group (*p* < 0.05). In all groups except the 12.5 mg/ml TF3 group, the WL rate and HYP release showed no significant difference between those in 30 s and 60 s (*p* > 0.05).

Comprehensive statistical analysis of the experimental results of WL rate and HYP release signified that 100 mg/mL was the best effective concentration of TF3 in biological modification, crosslinking, and anti-enzymatic hydrolysis. By contrast, whether the cross-linking time was 30 s or 60 s, the difference was not statistically significant. Therefore, 100 mg/ml TF3 crosslinking 30 s may be the most suitable concentration and time group for demineralized dentin biomodification crosslinking.

#### 3.2.2 SEM

Figure 3C shows the structure of demineralized dentin collagen in the control and ethanol groups collapsed and gaps appeared after enzymatic hydrolysis by Collagenase type I. However, after cross-linking with 5% GA and 100 mg/ml TF3 for 30 s, the dentin collagen structure and the fiber scaffold were stable. It was confirmed that TF3 cross-linked demineralized dentin collagen can improve the anti-enzymatic hydrolysis ability.

#### 3.2.3 TEM

**FIGURE 4:**
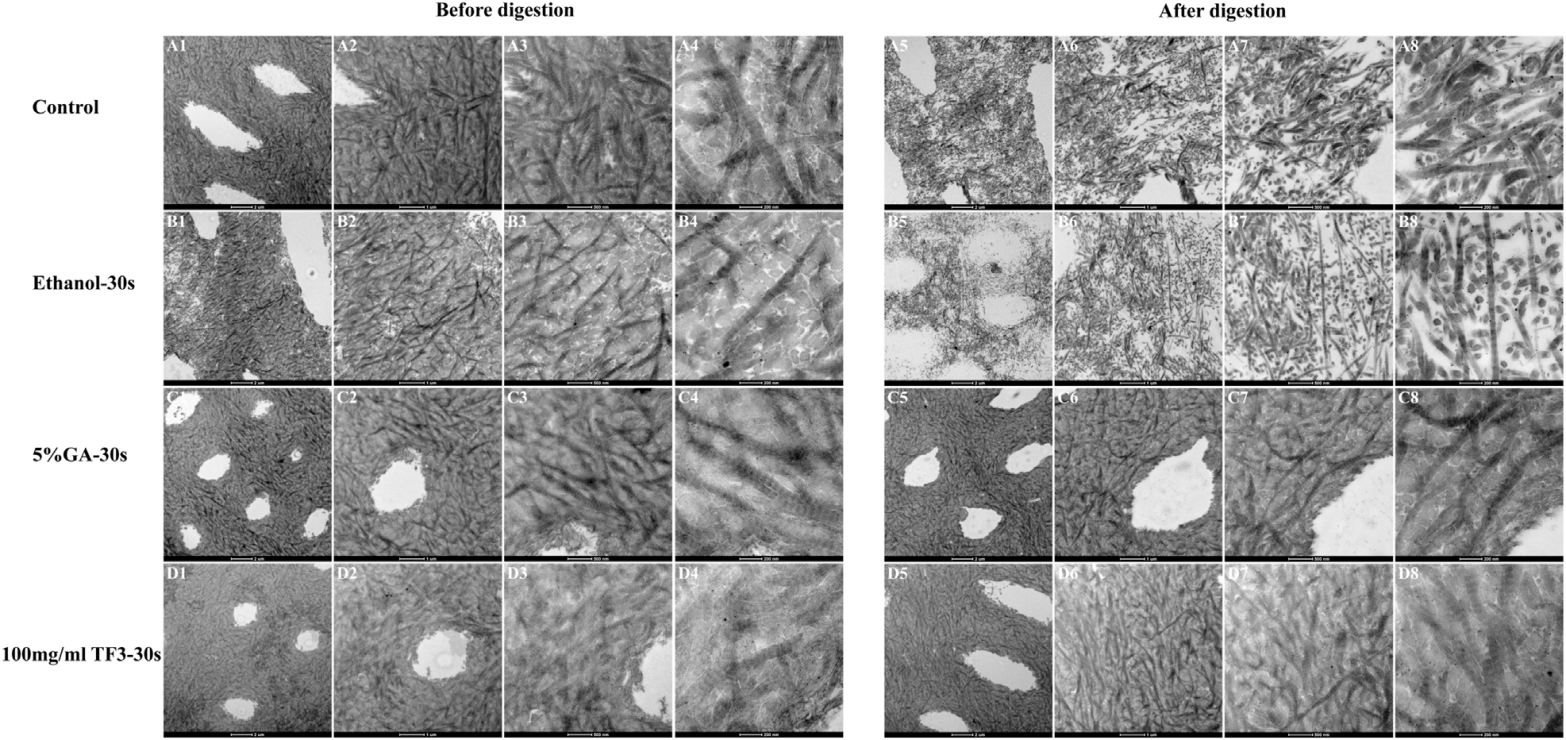
Representative TEM images of demineralized dentin matrix with diverse treatments. The scale bar for **(A1–D1)** and **(A5–D5)** is set at 2 um. The scale bar for **(A2–D2)** and **(A6–D6)** is set at 1 um. The scale bar for **(A3–D3)** and **(A7–D7)** is set at 500 nm. The scale bar for **(A4–D4)** and **(A8–D8)** is set at 200 nm. All the specimens were routine serial sections with a thickness of 4 μm, and 5 sections were obtained from each sample.

In Figure 4, there was a typical collagen-striated structure of the demineralized dentin collagen, the original fibers were arranged neatly and the gap was uniform. Compared with the control and ethanol group, after 5% GA and 100 mg/ml TF3 cross-linked for 30 s, the cross-striation structure of collagen became blurred and difficult to distinguish, indicating that the gap between collagen fibers was reduced through cross-linking, resulting in a more compact structure. Following 48 h of digestion with 0.1 mg/mL collagenase (Figure 4), the dentin collagen fibers in both the control and ethanol groups suffered significant damage, leading to their disintegration and the creation of gaps and voids. Some degraded collagen fiber fragments were scattered in gaps. In contrast, the collagen fibers in the GA and TF3 groups were densely arranged and the structure was intact, without gaps due to enzymatic hydrolysis.

### 3.3 Physicochemical properties

#### 3.3.1 Surface hardness test

**FIGURE 5:**
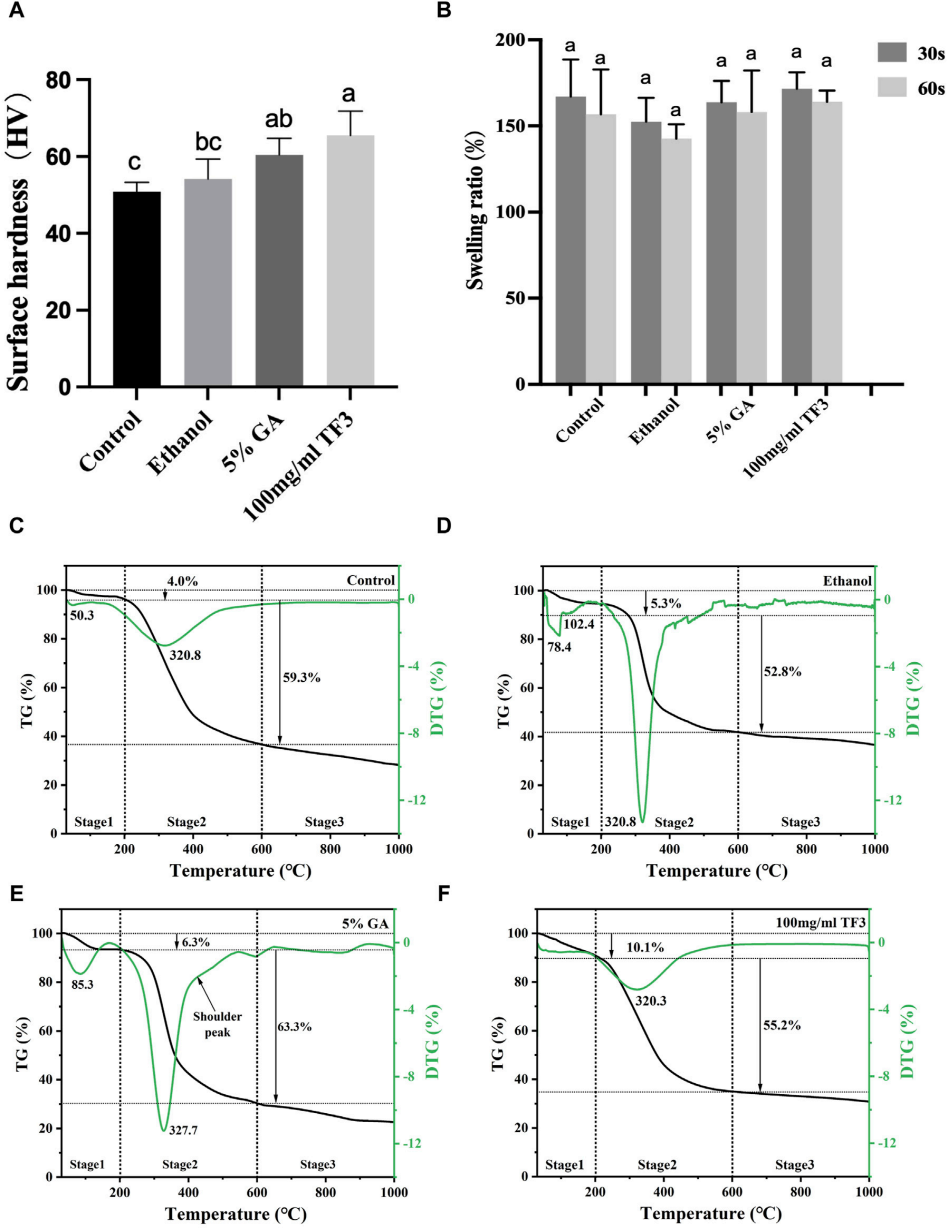
Surface hardness **(A)** and swelling ratio **(B)** of demineralized dentin matrix with diverse treatments. Variations in lowercase letters signify notable disparities among the groups (*p* < 0.05). TG-DTG of demineralized dentin matrix with diverse treatments **(C–F)**.

Displayed in Figure 5A are the measurements of surface hardness in collagen with varying treatments. Among all groups, the 100 mg/ml TF3 group exhibited the greatest surface hardness.

The 100 mg/ml TF3 group and 5% GA group marked a statistical difference from the control group (*p* < 0.05). No significant difference was shown in HV between the ethanol group and the control group (*p* > 0.05).

#### 3.3.2 Swelling ratio test

Figure 5B shows that the difference between the control group, the ethanol group, the 5% GA group, and the 100 mg/ml TF3 was not statistically significant differences when the cross-linking time was 30 s or 60 s (*p* > 0.05).

#### 3.3.3 TG-DTG

As shown in Figures 5C–F, the TG curve is segmental into three stages, with the initial stage (30°C–200°C) resulting from the depletion of surface-absorbed water, crystal water loss peak in demineralized dentin collagen and other volatile organic compounds. At this stage, the mass thermal decomposition loss rates of control, ethanol, GA, and TF3 were 4.0%, 5.3%, 6.3%, and 10.1%, respectively. The second stage (200°C–600°C) is the degradation peak of demineralized dentin collagen and the shoulder peak due to the different degrees of cross-linking. At this stage, the weight loss rates of the control, ethanol, GA, and TF groups were 59.3%, 52.8%, 63.3%, and 55.2%, respectively. The third stage (600°C–1,000°C) is the passive thermal region and the slow degradation of carbon-containing substances. In general, the weight loss rates of the control, ethanol, GA, and TF groups were 71.8%, 63.6%, 78%, and 69.3%, respectively, indicating that collagen cross-linking can affect the thermal stability of demineralized dentin collagen.

### 3.4 Enzyme inhibition

#### 3.4.1 *In situ* zymography

**FIGURE 6:**
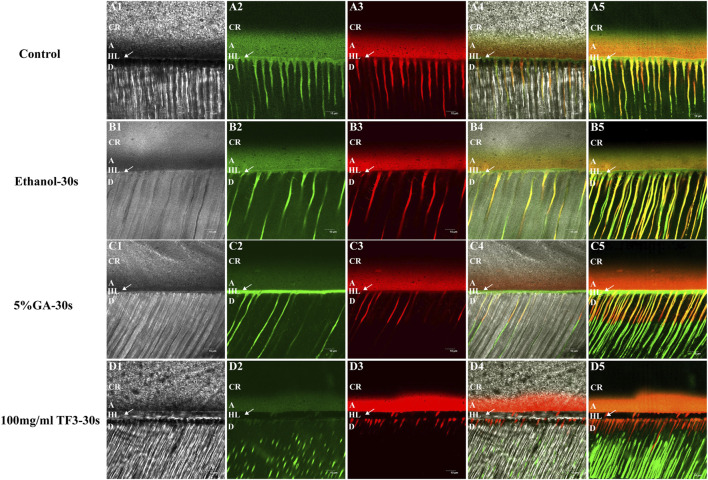
Representative confocal laser scanning microscopy images of the resin-dentin interface of demineralized dentin matrix with diverse treatments. **(A1–D1)** represent images under the light microscope. **(A2–D2)** represent the green fluorescence of MMPs. **(A3–D3)** represent the red fluorescence of the adhesive. **(A4–D4)** and **(A5–D5)** are the merged images. CR = Composite-Resin; A = Adhesive; HL = Hybrid Layer; D = Dentin. The arrow refers to HL. The scale bar is set at 10 um.

As shown in confocal laser scanning microscopy, the hybrid layer exhibited obvious green fluorescence within the 5% GA-treated, ethanol-treated, and non-treated control groups. This suggested significant hydrolysis of fluorescein-tagged gelatin by MMPs in the hybrid layer, with effective MMP activity, confirming that the control group, with 5% GA and ethanol, did not inhibit MMPs. In the hybrid layer of 100 mg/ml TF3 in the experimental group, there was almost no green fluorescence, indicating a significant suppression and negligible activity of MMPs, thus confirming the potent inhibitory impact of 100 mg/ml TF3 on MMPs. Representative images are shown in Figure 6.

### 3.5 Mechanism of cross-linking

#### 3.5.1 Fourier transform infrared spectroscopy

**FIGURE 7:**
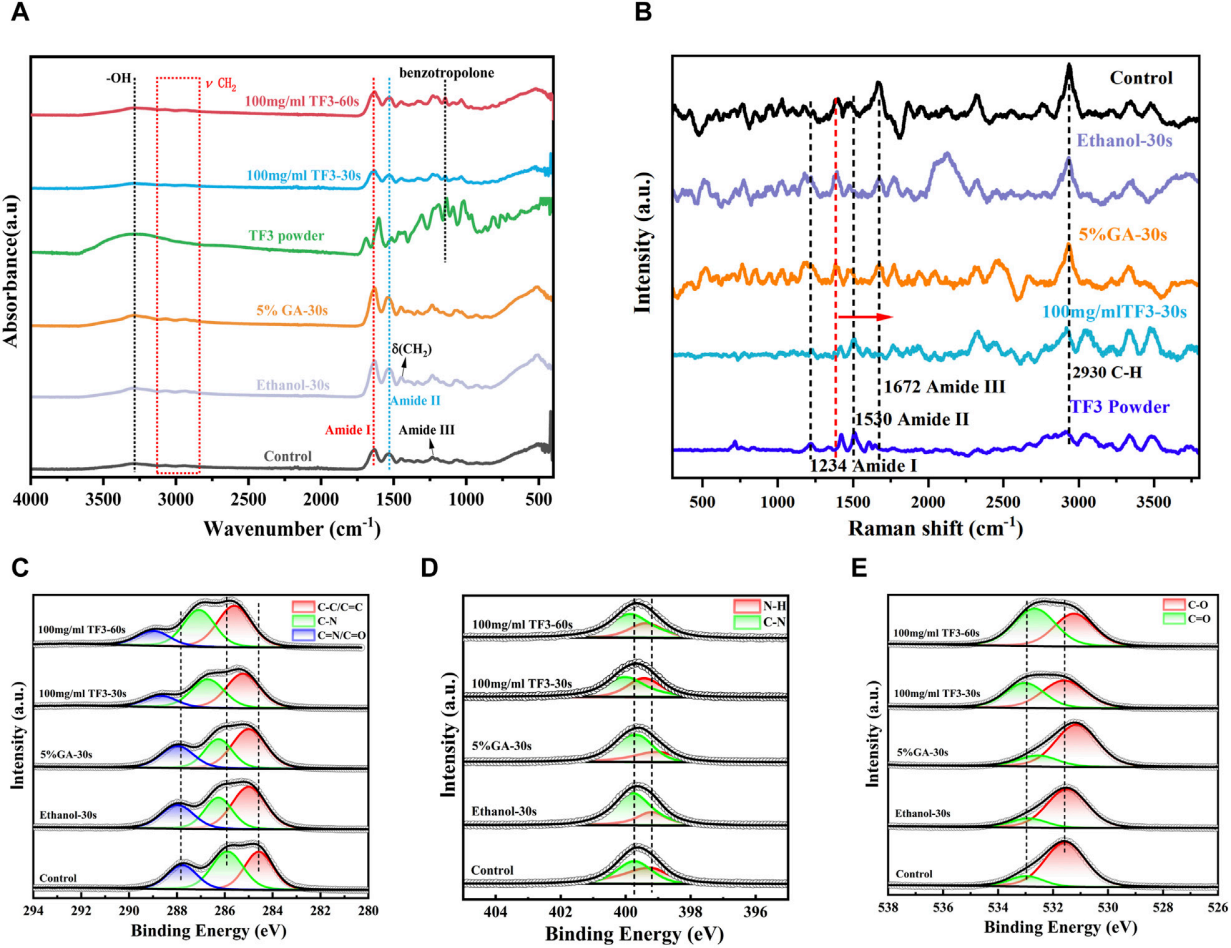
FTIR spectrum **(A)**. Raman spectrum **(B)**. XRD spectrum analysis **(C–E)**: Fine spectrum of C 1s **(C)**. Fine spectrum of N 1s **(D)**. Fine spectrum of O 1s **(E)**.

FTIR results (Figure 7A) showed several typical characteristic peaks before and after demineralized dentin collagen treatment: the absorption peak at 3,280 cm^−1^ was the -OH stretching vibration peak, representing amide A, the peaks at 2,940 and 3,060 were assigned to v (CH2) and amide B, respectively (Schmidt et al., 2020). The C=O stretching vibration at 1,633 cm^−1^ represents amide I; the heterogeneous combination of C-N extended vibration and N-H bending vibration at 1,535 cm^−1^ represents the amide II band. The absorption peak at about 1,450 cm^−1^ is caused by the shear vibration absorption of -CH2. At 1,235 cm^−1^, the combination of C-N vibration and N-H bending vibration represents the amide III band (Antonakos et al., 2007; Nisar et al., 2023). Compared with the untreated demineralized dentin collagen control, TF3 caused significant changes, including the absorption band of OH at 3,280 cm^−1^ at 30 s and 60 s of 100 mg/ml TF3 treatment became wider and the intensity increased. At the same time, the peak position shifted, and a more significant shift occurred at 60 s of 100 mg/ml TF3 treatment. In addition, a new and sharp absorption peak appeared in the cross-linked demineralized dentin collagen of TF powder and 100 mg/ml TF3 at 1,145 cm^−1^ (Liu et al., 2021). Compared with TF powder, the peaks of 100 mg/ml TF3 crosslinked for 30 s and 60 s shifted, and the intensity decreased, especially for 60 s.

#### 3.5.2 XPS

Figure 7C is the C 1s fine spectrum. The peaks at 284.8 eV, 285.9 eV, and 287.1 eV represent C-C/C=C, C-N, and C=N/C=O, respectively (Wang M. et al., 2023). The binding state of C element in different groups was diverse: in ethanol and GA groups, the binding energy of C element was relatively low, indicating that the interaction between C atom and other elements was weak; in the TF3 group, the binding energy of C element was higher, indicating that the interaction between C atom and other elements was stronger. This may be related to the hydrogen bond interaction between the phenolic hydroxyl group in TF3 and the C atom of demineralized dentin collagen (Liu et al., 2021). It is worth noting that with the extension of the action time, the binding energy became larger.

Figure 7D is the N 1s fine spectrum. The peaks at 399.2 and 399.7 eV are associated with N-H and C-N bonds, respectively (Wang M. et al., 2023). The peak position of the ethanol group was close to that of the control group, indicating that the chemical structure of the demineralized dentin collagen was not influenced by the ethanol. In the GA group, the N-H bond slightly shifted to the low binding energy direction, indicating that GA interacted with the N atoms in the demineralized dentin collagen. In the TF group, the peaks of N-H bond and C-N bond shifted significantly in the direction of high binding energy, indicating that TF3 could significantly change the chemical environment of demineralized dentin and affect its molecular structure.

Figure 7E is the O 1s fine spectrum. The peaks at 531.2 eV and 532.6 eV are referred to C-O and C=O, respectively (Wang M. et al., 2023). The chemical state of O in different groups was also significantly different: the peak position of ethanol was close to that of control, indicating that ethanol did not change the chemical structure of demineralized dentin. In the GA group, the C=O double bond did not change significantly, and the C-O bond shifted slightly to the direction of low binding energy, indicating that GA could interact with O atoms in demineralized dentin collagen (Gai et al., 2023). In the TF3 group, the peaks of C-O bond and C=O bond were significantly shifted to the direction of high binding energy. With the prolongation of TF3 treatment time, the shift was more obvious, indicating that TF3 could significantly change the chemical structure of demineralized dentin. More importantly, TF3 can significantly increase the content of C=O bond, indicating that TF and demineralized dentin collagen may form ester bonds through covalent interaction (van den Brand et al., 2004).

#### 3.5.3 Raman spectroscopy

As depicted in Figure 7B, the characteristic peaks at 1,672, 1,530, and 1,234 cm^−1^ are the typical characteristic peaks of the three-dimensional spiral structure of collagen fibers (Xu and Wang, 2012). These peaks were associated with the amide I band, amide II band, and amide III band, respectively, and exhibited sensitivity to the molecular conformation of the polypeptide chain, aligning with the results of infrared spectroscopy (Antonakos et al., 2007; Nisar et al., 2023). The peak at 1,450 cm^−1^ and 2,930 cm^−1^ referred to the C-H bond, reflecting the organic components present in demineralized dentin (Lazar et al., 2005).

The control, ethanol, and GA groups showed similar peaks, indicating that ethanol and GA treatments did not induce significant structural changes in demineralized dentin collagen. However, in the TF3 group, differences emerged compared to the control. Specifically, the amide I, II, and III bands appeared slightly wider, with the amide I band peak experiencing a slight reduction in intensity and a shift towards the lower band region. Conversely, the amide II band peak exhibited an increase in intensity. For the peak of collagen peptide chain C-H bond at 1,450 cm^−1^, TF3 shifted to the high band area. These observations suggest a robust cross-linking reaction between TF3 and the carboxyl, hydroxyl, and amino groups present in demineralized dentin collagen.

## 4 Discussion

Theaflavins (TFs) are natural compounds in black tea, which are a general term for a class of benzotropolone compounds containing multiple hydroxyl or phenolic hydroxyl groups. TFs are mainly composed of four dimers, namely, TF1, TF2A, TF2B and TF3, which are different due to the number and location of gallic acid groups. Among them, TF3 contains two gallic acyl groups, which is the most abundant and most active component (Hass et al., 2021). Current research indicates that TFs are capable of rapidly crosslinking demineralized dentin collagen film, safeguarding it against collagenase degradation, and its effect is equivalent to or better than PA (Liu et al., 2021). The reason lies in the structural difference between TFs and PA. In contrast to the PA dimer, where two catechins are connected by a single C-C bond, the TFs feature two catechin units linked by a benzotropolone skeleton. This unique structure incorporates a carbonyl group positioned near the hydroxyl group. The carbonyl group’s ability to form hydrogen bonds with hydrogen donors, such as hydroxyl or amino groups, facilitates the cross-linking of collagen. In addition, TFs have aromatic ring structures, abundant in phenolic hydroxyl groups and gallic acyl groups, which have also been shown to make a crucial contribution to collagen cross-linking (Liu et al., 2013; Vidal et al., 2016). Among the four dimers of TFs, in addition to the same structure, such as benzophenone skeleton and carbonyl group, TF3 has the most phenolic hydroxyl and galloyl groups, which is speculated to have the most crosslinking potential (Truong and Jeong, 2021). However, there are still no studies reporting TF3’s cross-linking impact on demineralized dentin collagen. Hence, in this study, we used TF3 as a novel cross-linker and investigated its cross-linking efficacy and mechanism. The results showed that TF3 can effectively cross-link dentin collagen and enhance its enzymatic resistance and biomechanical properties within the clinical time, mainly through hydrogen bonding.

First, we performed theoretical calculations and used molecular docking to predict the interaction between TF3 and type I collagen. The results indicated that the binding energies of TF3 with type I collagen structure 1QSU, 4OY5 and 1CGD were −5.8, −6.1 and −5.4 kcal/mol, respectively, indicating that they could spontaneously bind. This is similar to the predicted result of other studies (Vaidyanathan et al., 2007) using molecular docking to simulate the binding energy between 10-methacryloxy decamethylene phosphate and demineralized dentin type I collagen between −4.5 and −8.9 kcal/mol, which also provides evidence for the interaction between TF3 and collagen. Predictions by MD (Table1) show that TF3 and type I collagen can be combined through various forces, including hydrogen bonds, hydrophobic interactions, and water bridges, among which hydrogen bonds are the most important. For example, the hydrogen bond formation frequency between TF3 and GLU73 in 1QSU structure is as high as 136%, forming two hydrogen bonds with a frequency of 68%. This means that TF3 can spontaneously bind to the active sites of collagen through hydrogen bonds, thereby achieving cross-linking of demineralized dentin collagen. However, it should be noted that the simulation may not fully reflect the real interaction (Bertassoni et al., 2012).

Next, to study the ability of TF3 cross-linked demineralized dentin collagen to resist exogenous collagenase digestion, this study used 0.1 mg/mL exogenous type I bacterial collagenase for collagenase digestion experiment (Liede et al., 1999). Notably, the concentration level of collagenase is two to six orders of magnitude greater than those in human oral fluid (Paivi et al., 2002). Firstly, the overall understanding of the protective relationship of TF3 on dentin collagen was obtained by drying weight loss experiment. This experiment is based on the fact that there is a balance between the degradation rate of soluble dentin collagen small molecule peptides or amino acids and their release rate from the insoluble demineralized dentin matrix that acts as a small molecular sieve (Garnero et al., 2003; Takahashi et al., 2013). So drying weight loss can indirectly evaluate the enzymatic hydrolysis tolerance of demineralized dentin collagen to exogenous type I bacterial collagenase at the overall level. Secondly, given that dentin type I collagen comprises roughly 10% hydroxyproline, the majority of other proteins have minimal or no similar amino acid composition (Marshall et al., 1997). The ability of dentin collagen to withstand collagenase digestion can be specifically detected by measuring hydroxyproline release.

As depicted in Figures 3A,B, WL and HYP release of TF3 were notably less compared to the control and ethanol group, and decreased with the increase in concentration, which was concentration-dependent. Notably, the effect of 100 mg/ml TF3 cross-linking was even better than that of 5% GA, and there was almost no difference in weight loss and hydroxyproline release between 30 s and 60 s. This may be related to the fact that high concentrations of TF3 can penetrate the collagen network more quickly.

In addition, we also detected the impact of TF3 on the activity of endogenous proteases by *in situ* zymography. MMPs exist in the mineralized dentin matrix in the form of zymogens, capable of being activated through acid etching and hydrolyzing the exposed collagen (Mazzoni et al., 2013). The experimental results revealed a prominent green fluorescence at the base of the hybrid layer and within the dentinal tubules in the control group (Figure 6A5), the ethanol group (Figure 6B5), and the GA group (Figure 6C5), signifying that the activity of endogenous dentin MMPs in these regions was uninhibited (Mazzoni et al., 2012). In contrast, there was black at the base of the hybrid layer and within the adjacent dentinal tubules in the TF3 group (Figure 6D5), and no green fluorescence was detected. This suggests that TF3 effectively inhibited the activity of endogenous MMPs. In addition, this also explains the reason why TF3 can effectively resist collagenase digestion, that is, TF3 may interact with the enzyme, inactivate the C-terminal peptidase, and interfere with the binding of collagenase to collagen (Sabatini and Pashley, 2015). In another part of our research on the interaction between TF3 and enzymes, molecular docking simulation showed that the binding energy of TF3 and MMPs was −7.3 ∼ −8.7 kcal/mol, and the binding energy of TF3 and cysteine protease was even −9.3 ∼ −10.0 kcal/mol. Therefore, it is reasonable to believe that TF3 can inhibit the activity of endogenous proteases.

To further study the impact of TF3 on maintaining the structure of demineralized dentin collagen, we conducted observations using SEM and TEM. The SEM results (Figure 3C) indicate that, compared to the control group and the ethanol group, the surface structure of collagen in the 5% GA and 100 mg/ml TF3 groups appears smoother and denser before enzymatic hydrolysis. However, after enzymatic hydrolysis, the surface becomes slightly rough, yet the collagen structure remains intact, and both intertubular and peritubular dentin remain viable. The TEM results (Figure 4) reveal that the characteristic cross-striation structure of collagen in the 5% GA and 100 mg/ml TF3 groups becomes thicker and blurred. The collagen fibers remain dense and intact with characteristic cross-striations even after enzymatic hydrolysis. These findings corroborate the results of WL and HYP release, suggesting that 100 mg/ml TF3 possesses a crosslinking efficiency similar to that of 5% GA. Both agents can crosslink with dentin collagen and reduce its spacing to maintain structural integrity, thereby enhancing its resistance to enzymatic hydrolysis. Consequently, the first and second null hypotheses were dismissed.

Reports indicated that the surface hardness of demineralized dentin is an important index affecting the bonding durability (Hardan et al., 2022). During the process of dentin bonding, collagen possesses a robust surface, which can resist collagenase digestion and more effectively prevent collagen from collapsing post acid etching-washing, thereby enhancing adhesive penetration (Scheffel et al., 2014). This research focused on testing the surface Vickers hardness of demineralized dentin collagen post-crosslinking. The results showed that (Figure 5A), after crosslinking with ethanol, 5% GA, and 100 mg/ml TF3, the demineralized dentin surface of the 100 mg/ml TF3 group had the highest Vickers hardness, which may be related to the crosslinking effect to shorten the distance between collagen molecules and make its structure denser (Bedran-Russo et al., 2008).

Additionally, collagen’s hydrophilicity is influenced by cross-linking agents and the swelling rate is positively correlated with hydrophilicity (Leme et al., 2015). In general, a heightened level of cross-linking between collagen fibers and a decrease in bound water decreases the swelling rate (Miles et al., 2005). However, the study (Figure 5B) revealed no significant difference (*p* > 0.05) among the groups. This suggests that 100 mg/ml TF3 did not change the hydrophilicity of the dentin matrix, also, the inter-collagen fiber-bound water was not reduced during the treatment time (30 s/60 s), which we speculate may be related to the fact that TF3 is soluble in water and the shorter treatment time. Our experimental results were different from those of some studies (Deng et al., 2013; Hiraishi et al., 2013), which showed GA solution’s efficacy in diminishing the swelling rate of demineralized dentin matrix, probably because their experimental time (12 h) was much longer than ours (30 s/60 s).

The TG-DTG experiment was conducted to evaluate the thermal stableness of the dentin matrix after cross-linking modification. The denaturation temperature of the dentin matrix is affected by the form, quantity, distribution, and water content of the crosslinking agent. Considering that high temperatures have the potential to disrupt protein-water interactions, break hydrogen bonds, and cause the evaporation of bound water, thermal stability serves as an indicator of a protein structure’s resistance to thermal reactions (Rochdi et al., 1999). The second stage of TG-DTG (200°C–600°C) showed a peak of demineralized dentin collagen carbonization degradation and a shoulder peak due to different cross-linking degrees. At this stage, the weight loss rates of the control, ethanol, GA, and TF groups were 59.3%, 52.8%, 63.3%, and 55.2%, respectively. TF3’s depletion rate was less than the control group, suggesting that TF3 improved the structural stability and reduced the loss rate of mass thermal decomposition by crosslinking with demineralized dentin. In the GA group at this stage, the main peak of the DTG curve shifts towards the high-temperature region, and a shoulder peak appears at 430°C beyond the main peak at 327.7°C. Additionally, the weight loss rate is relatively high, which may be related to factors such as uneven crosslinking of collagen, multiple thermal decompositions, and additional thermal weight loss caused by the decomposition of the crosslinking agent (Miles et al., 2005).

The results of surface hardness and TG-DTG tests revealed that the biomechanical characteristics of demineralized dentin collagen cross-linked with TF3 have been enhanced. Consequently, the third null hypothesis was likewise dismissed.

To study the mechanism of action of TF3 and type I collagen, we used FTIR, XPS, and Raman spectroscopy. In FTIR detection, the maintenance of amide I, II, and III peaks in TF3 group indicated that TF3 did not disrupt the triple helix structure of dentin collagen. The broadening of amide I (∼1,660 cm^−1^) observed in Figure 7A could be caused by hydrogen bonding interactions between the amino and amide groups of collagen and the phenolic hydroxyl groups of TF3 (He et al., 2011). After TF3 treatment of demineralized dentin collagen, the OH absorption peak at 3,280 cm^−1^ shifted, the absorption band widened and the intensity increased, indicating the formation of intermolecular hydrogen bonds. To further confirm the existence of hydrogen bonds, we increased the cross-linking time of TF to 60 s. The results showed that the absorption peak of OH at 3,280 cm^−1^ shifted more obviously, the absorption band was wider and the intensity increased more obviously (Figure 7A), which confirmed that TF and demineralized dentin collagen formed intermolecular hydrogen bonds. In addition, the ratio of A1235/A1450 at the amide III bond to 1450 cm^−1^ is also an important criterion for evaluating the structural integrity of the collagen triple helix. The collagen A1235/A1450 with complete triple helix structure is about 1 (Figueiró et al., 2006) and the principle of denatured gel is less than 0.6 (Sylvester et al., 1989; Figueiró et al., 2006). In this experiment, the A1235/A1450 of the control group, the ethanol group, the GA group, and the TF3 group were about 1.07, 0.86, 0.97, and 0.66, respectively, all between 0.6-1.0. It also shows that TF3 has a strong interaction with demineralized dentin matrix collagen molecules without changing the triple helix structure. The reason for the decrease of A1235/A1450 caused by TF3 may be that the -CH2 in its structure significantly increases the absorption spectrum at ∼ 1,450 cm^−1^, while the amide III bond at ∼ 1,235 cm^−1^ is not strengthened. In addition, in the TF powder and 100 mg ml TF3 group, the C-C (-OH) stretching vibration band at 1,145 cm^−1^ attributed to the benzotropolone structure of TF3 changed, indicating that the benzotropolone structure also played a role in the cross-linking between TF3 and demineralized dentin collagen, which was also verified in the study of Liu’s (Liu et al., 2021). When analyzing the reason why the cross-linking effect of low-concentration TF3 is stronger than that of PA, it is speculated that the additional fused ring and/or a great many phenolic hydroxyl groups of benzotropolone in TF3 may have potential influence.

In XPS detection, the peaks at 284.8 eV, 285.9 eV and 287.1 eV in the fine spectrum of C 1s (Figure 7C) represent C-C/C=C, C-N, and C=N/C=O bonds, respectively. The binding energy of ethanol and GA groups is relatively low, while the binding energy of the TF3 group is higher, which is related to the hydrogen bond interaction between phenolic hydroxyl groups in TF3 and demineralized dentin collagen (Gilbert et al., 2013). In the N 1s and O1s fine spectrum (Figures 7D,E), the N - H bond (399.2 eV), C - N bond (399.7 eV), C - O bond (531.2 eV), and C = O bond (532.6 eV) wave peaks were close in position in the ethanol and control groups. In contrast, all of these wave peaks in the TF group were significantly shifted to the high binding energy direction, indicating that the structure of demineralized dentin collagen may be affected by TF3 in molecular aspect. More importantly, the content of C=O bond in the TF3 group increased significantly, suggesting that TF3 and demineralized dentin collagen may form ester bonds through covalent interaction (Nosenko et al., 2015). At the same time, it also indicates the formation of intermolecular hydrogen bonds between TF3 and demineralized collagen to a certain extent.

In the Raman spectroscopy (Figure 7B), the characteristic peaks of 1,672, 1,530 and 1,234 cm^−1^ represent the amide I, II and III bands of the three-dimensional spiral structure of collagen fibers, respectively. The control, ethanol and GA groups showed similar peaks, while in the TF3 group, the amide I band, II band and III band were slightly widened, the amide I band peak was slightly reduced and slightly shifted to the low band region, and the peak of the amide II band was increased, indicating that TF3 preserved the integrity of the dentin collagen triple helix structure (Nosenko et al., 2015). This finding is consistent with the results of FTIR. In addition, in the TF3 group, the peak at 1,450 cm^−1^ representing the C-H bond of the collagen peptide chain shifted to the high-band region, indicating that TF3 had a strong cross-linking reaction with the carboxyl, hydroxyl, and amino groups of the demineralized dentin collagen (Rangan et al., 2020).

FTIR, XPS and Raman studies have shown that TF3 can cross-link demineralized dentin collagen through hydrogen bonds, covalent interactions, etc., further providing strong evidence that TF3 can cross-link demineralized dentin collagen. Consequently, the initial incorrect hypothesis is once more dismissed.

In summary, this study confirmed for the first time that TF3 can efficiently cross-link demineralized dentin collagen, significantly improve its resistance to endogenous and exogenous collagenase digestion, and improve its biomechanical properties. These experiments lay the foundation for the clinical application of TF3 and have very important significance. However, its effectiveness in improving the durability of dentin bonding has not been confirmed in the study, which is slightly regrettable. In subsequent experiments, we will further optimize and study the effect of TF3 on dentin bonding properties, including immediate and durable dentin bonding strength, nanoleakage, fracture mode, *in situ* zymography, etc.

## 5 Conclusion

Our research revealed that TF3 is capable of efficiently cross-linking demineralized dentin collagen through hydrogen bonds and covalent interactions in clinical time, resisting collagenase digestion, and improving biomechanical properties. At the appropriate treated concentration and time, the safeguarding function of TF3 on dentin collagen is similar to or even higher than that of GA, which lays a foundation for the subsequent study of the effect of TF3 on dentin and also provides a novel possibility for enhancing the longevity of the adhesive.

## Data Availability

The original contributions presented in the study are included in the article/Supplementary Material, further inquiries can be directed to the corresponding authors.
