# Supplementary material for: Theaflavin −3,3'-digallate/ethanol: a novel cross-linker for stabilizing dentin collagen
Source: Front Bioeng Biotechnol. 2024 May 15;12:1401032. doi: 10.3389/fbioe.2024.1401032 (PMC11133682; doi:10.3389/fbioe.2024.1401032)
Supplement: Supplementary file 1 [file Presentation1.PDF]

Subject informed consent

Project Name: The effect and mechanism of theafluorotine-3,3’-digallate on dentin bonding.
Version number and version date: February 18, 2023
Informed consent Version number and version date: February 18, 2023

Dear patients,

We invite you to participate in the study on the effect and mechanism of theafluorotine-3,3’-digallate on dentin adhesion approved by The Stomatology Hospital affiliated to Guangxi Medical University. The study will be conducted at the Stomatology Hospital affiliated to Guangxi Medical University, and 800 patients are expected to volunteer. This study has been reviewed and approved by the Ethics Committee of Stomatology Hospital affiliated to Guangxi Medical University.

This notice will provide you with some information to help you decide whether to participate in this clinical study. Your participation in this study is completely voluntary, and your decision will not affect your normal treatment rights and benefits in our hospital. Please rest assured! If you choose to participate in this study, our research team will do our best to ensure your safety and interests during the study!

Please read these instructions carefully, and if you have any questions, please contact the researcher responsible for explaining informed consent to you.

Research Background: With the development of society and the improvement of living standards, there is an increasing demand for minimally invasive oral aesthetic repair, such as resin filling and all-porcelain restoration. Dentin bonding is the basis of minimally invasive oral aesthetic repair and an indispensable treatment in oral clinic. So far, however, we have had great difficulty and challenges in sticking. Therefore, how to improve the durability of dentin bonding is not only a hot research topic, but also a clinical problem to be solved urgently.

At present, there are two ways of dentine bonding, namely total acid etching and automatic acid etching. Regardless of total acid etching or self-acid etching, the adhesive can not completely penetrate into the depth of tooth acid etching and demineralization, which is also the main reason for the lack of durability. At present, there are many strategies and methods to improve the durability of dentin adhesion to a certain extent, but there are still some problems such as biosafety, cumbersome operation, and long-term effectiveness is uncertain, so there is no consensus in the field of dentin adhesion.

In view of this, scholars are still working on finding better ways to improve the durability of the bond. In recent years, studies on theaflavins are increasing due to their anti-cancer, antibacterial, anti-inflammatory and other biological activities. Theaflavins, known as "tea gold", are a group of compounds with benzodiazephenone structure, mainly composed of theaflavins (TF1), theaflavin-3-gallate (TF2A), theaflavin-3 '-gallate (TF2B) and theaflavin-3, 3' -bis gallate (TF3).In the field of oral cavity, studies have shown that it has the effect of anti-caries, prevention of periodontal disease and oral cancer. In addition, different studies have shown that phenolic hydroxyl, gallic group, benzodrophenone and other structures have collagen crosslinking. Therefore, in combination with the structure-activity relationship, molecular weight and the role and mechanism of natural polyphenols in dentin bonding, this study took theafluorotine-3,3'-digallate as the research object, carried out preliminary research on it, and preliminarily confirmed that it has good collagen cross-linking and anti-enzymatic hydrolysis. Therefore, this research

group intends to take theafluoroflavin-3,3'-digallate as the research object to study its biological modification effect on demineralized dentin and its mechanism, as well as its immediate and durable impact and action mechanism on dentin adhesion, in order to construct a new material or method for dentin adhesion, provide scientific basis for improving the strength and durability of dentin adhesion, and provide a feasible new scheme for clinical practice.

Research objective: To study the mechanism, physicochemical properties and anti-enzymatic hydrolysis ability of theafluorotine-3,3'-digallate (TF3) on the biological modification of demineralized dentin, to study the immediate and durable application effect of TF3/ ethanol on dentin adhesion, and to explore its clinical application, in order to build a new material or method for dentin adhesion, to provide a scientific basis for improving the strength and durability of dentin adhesion, and to provide a feasible new scheme for clinical practice.

Subjects: Patients in the Department of Oral and maxillofacial Surgery, Stomatology Hospital affiliated to Guangxi Medical University, who needed to have their third molars removed for disease prevention or treatment.

Research process: Third molars that needed to be removed due to impacted or orthodontic conditions were collected from the Department of Oral and maxillofacial Surgery and general outpatient department of Stomatology Hospital affiliated to Guangxi Medical University. Immediately after removal, wash and disinfect, store in 0.1% thymol solution at 4° C, and use within 1 month. This study intends to take theafluorotine-3,3'-digallate as the object of study to thoroughly study the biological modification effect and mechanism of theafluorotine-3,3'-digallate on demineralization dentin, its immediate and durable impact on dentin adhesion and the mechanism of action, so as to establish a new material or method for dentin adhesion, provide scientific basis for improving the strength and durability of dentin adhesion, and provide a feasible new scheme for clinical practice.

Possible risks and discomfort: This study uses your discarded specimens and does not cause any possible risks and discomfort

Expected benefits: To study the effect and mechanism of theafluorotine-3,3'-digallate on dentin adhesion, provide scientific basis for improving the strength and durability of dentin adhesion, and provide a feasible new scheme for clinical practice.

Free treatment: This study does not affect your treatment plan and treatment process, and the treatment fee will not change.

Compensation: This study does not affect your treatment plan and treatment process, so you will not be compensated for participating in this study.

Compensation: None

Confidentiality: Your personal information records will be properly kept and treated as

confidential, and will only be provided in accordance with the law when accepting the supervision of relevant departments (ethics committee, food and Drug Administration), and will not be disclosed to third party individuals or institutions. At the end of the study, your dental samples will be destroyed. Any information transmitted electronically will be renamed to ensure that Confidentiality of information. All computer information will be password protected. The results of the study may be reported at medical conferences and published in scientific journals. But any information that identifies you personally will not be used.

Regain informed consent: none

Voluntary: You have the right of free participation and withdrawal, informed consent. Even if participation in the trial is voluntary, there will be no loss of equity or penalty for withdrawal from the trial. If you participate in the study, you can withdraw at any time without any reason. Whatever your decision is, it will not affect your normal treatment or your relationship with your healthcare staff.

Subject obligation: If you are requested to truthfully provide information about your physical condition, cooperate and respect the researcher.

Contact information: If you have any questions related to this study, or have any discomfort or injury during the study, or have any questions about the rights and interests of participants in this study, you can contact the researcher Zhiyong Chen at 18260970966.

If you have any questions or complaints about the researchers during the study, you can contact the Ethics Committee of The Affiliated Stomatology Hospital of Guangxi Medical University at 0771-2387994 or email gxmucsethic@163.com.

Subject signature page

Subject consent statement:

□ I have read the above introduction of this study, and the research doctor has explained the research content to me in detail. I have no more questions about this study to consult before signing the informed consent. On this basis, I voluntarily participate in the clinical study described in this article, and my decision is based on full understanding of the possible risks and benefits of participating in this study. In addition, the researcher did not use deception, inducement or coercion to force me to agree to participate in the study, and I knew that I could withdraw from the study unconditionally at any stage.

□ Due to the subject's incapacity or limited capacity, this informed consent shall be signed by his/her guardian or legal agent.

Signature of subject: Signature of legal representative:

Date: Date:

Subject contact Information: Legal representative Contact Information:

Signature of Guardian: Signature of Fair Witness:

Date: Date:

Guardian Contact: Fair Witness Contact:

Researchers state:

I confirm that I have explained to the patient the details of the study, in particular the possible risks and benefits of participating in the study.

Investigator signature:

Date:

Contact Information:

Note: This page is the subject's signature page, where the study doctor will explain the study content and relevant information to the subject in detail. Informed consent shall be signed by the subject himself/herself/guardian/legal representative and the study doctor who will explain to the subject. If the subject has any questions about the study, the investigator should immediately explain them in detail to the subject in person. After signing, both the investigator and the subject will keep one original copy.
